# GAN-Based Approach for Diabetic Retinopathy Retinal Vasculature Segmentation

**DOI:** 10.3390/bioengineering11010004

**Published:** 2023-12-21

**Authors:** Anila Sebastian, Omar Elharrouss, Somaya Al-Maadeed, Noor Almaadeed

**Affiliations:** Computer Science and Engineering Department, Qatar University, Doha P.O. Box 2713, Qatar; elharrouss.omar@gmail.com (O.E.); s_alali@qu.edu.qa (S.A.-M.); n.alali@qu.edu.qa (N.A.)

**Keywords:** diabetic retinopathy, retinal blood vessel segmentation, GAN, fundus images, deep learning

## Abstract

Most diabetes patients develop a condition known as diabetic retinopathy after having diabetes for a prolonged period. Due to this ailment, damaged blood vessels may occur behind the retina, which can even progress to a stage of losing vision. Hence, doctors advise diabetes patients to screen their retinas regularly. Examining the fundus for this requires a long time and there are few ophthalmologists available to check the ever-increasing number of diabetes patients. To address this issue, several computer-aided automated systems are being developed with the help of many techniques like deep learning. Extracting the retinal vasculature is a significant step that aids in developing such systems. This paper presents a GAN-based model to perform retinal vasculature segmentation. The model achieves good results on the ARIA, DRIVE, and HRF datasets.

## 1. Introduction

Diabetes is a global health concern found among individuals of different age groups. Diabetic retinopathy (DR) is a condition of the eyes that might occur in persons who suffer from diabetes for a very long period. DR patients will have certain DR lesions behind their retinas. Fundus photographs of patients which are taken with the help of fundus cameras are used by ophthalmologists to diagnose DR. Manually examining these fundus images requires a lot of time and is also error-prone. Moreover, there has been an alarming increase recently in the number of diabetes patients. Due to this, the limited number of ophthalmologists available to carry out this procedure is becoming a barrier to the timely diagnosis of DR [1]. Early detection of DR is of great importance since this will help give timely treatment to patients to avoid any undesirable consequences that may occur as a result of DR progressing to a severe stage. Several computer-aided automated systems are being developed to address this issue using techniques like deep learning.

**Figure 1:**
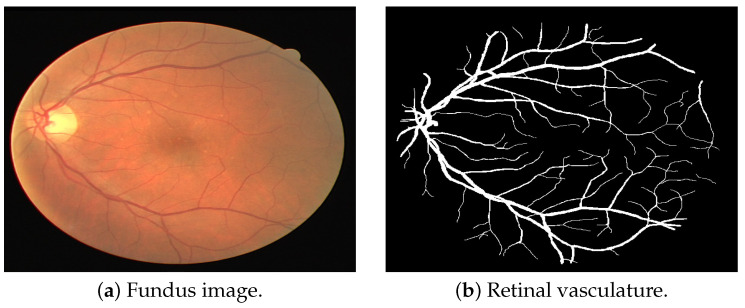
Retinal vasculature segmentation sample [5].

Retinal vasculature segmentation has a significant role in developing such systems. It is usable as a pre-processing/feature extraction step to develop DR detection or DR grading systems [2]. Also, it is useful for treating and detecting the risk of many diseases like diabetes mellitus, hypertension, cardiovascular disease, etc. [3]. Due to this importance, many new methods are used to perform this task. But, there are several challenges in achieving this efficiently and accurately. One major problem is the unavailability of sizeable datasets. The state-of-the-art datasets used for retinal vasculature extraction are small in size. This forces some studies to use a combination of multiple datasets collected under different settings and taken using different cameras [4]. Another major challenge is the wide disparity in the thickness of the retinal blood vessels. Hence, the utilized retinal vessel extraction methods should be capable of efficiently segmenting both thick and thin vessels. Yet another challenge is that retinal features differ from patient to patient. Finally, other structures in the retina, like the Optic Disc, Fovea, and DR lesions, may be wrongly detected as retinal blood vessels. Hence, in this study, we put forward a Pix2Pix GAN model, which can overcome all these challenges and segment retinal blood vessels efficiently on multiple datasets, including ARIA, DRIVE, and HRF datasets. An example of manually extracted retinal vasculature from an image of the DRIVE dataset is presented in Figure 1.

## 2. Related Works

Retinal vasculature extraction is a challenging task of extracting retinal blood vessels of varying thicknesses from fundus images effectively. This resulting vasculature should be free from other structures present in the retina. Also, the method used should be able to perform the same on fundus images collected under different settings. Over the years, researchers have used several traditional and artificial intelligence-based techniques to achieve this [6,7,8]. Artificial intelligence practices like machine learning and deep learning are the most highly preferred techniques for this task.

### Diabetic Retinopathy Retinal Vasculature Segmentation

Several authors have explored the use of both traditional and machine-learning techniques for retinal vasculature extraction. For instance, the researchers in [9] made use of contrast-limited adaptive histogram equalization for enhancing the retinal images’ contrast, followed by mathematical morphology to reduce noise. The fuzzy c-means method was used for blood vessel extraction, and further refinement was achieved through an integrated level set approach. Fan et al. [10] used the image matting technique for retinal vasculature extraction. They automatically generated a trimap using the region features of the vessels. Later, hierarchical image matting was used for extracting the pixels of blood vessels present in unfamiliar regions. A three-stage algorithm for retinal vasculature extraction was introduced by the authors in [11]. Initially, binary images were extracted by preprocessing the green plane of the input images, and larger vessels were identified from these. After this, a Gaussian mixture model classifier was used for classification, and, in the third stage, the classified output from the previous stage was combined with significant portions of the blood vessels. Hossain and Reza [12] proposed a model for detecting blood vessels using the Markov Random Field method. They found the energy of clique sets using Markov–Gibbs equivalence. Finally, they utilized the Bayesian rule to determine the joint distribution.

Researchers have effectively utilized deep learning architectures. For instance, the authors in [13] employed a Convolutional Neural Network (CNN) for generating a vessels probability map, which helped to distinguish vessels as well as background pixels in low-contrast regions. Further, a fully connected Conditional Random Field (CRF) was used with the vessel probability map to achieve better segmentation accuracy. In a different study, the authors in [14] introduced a segmentation technique using a fully connected CNN with pre/post-processing. The final steps helped with noise removal and to obtain fine segmentation results.

A two-stage approach for vessel extraction was introduced by the authors in [15]. The first one utilized a CNN to correlate the image patch and the ground truth. In the next one, a visual codebook was formed by propagating the training patches in the CNN, allowing feature vectors to query this search space. Additionally, Sine-Net, a deep CNN-based architecture, was used for blood vessel segmentation [16]. It made use of up-sampling and down-sampling for capturing features of vessels with different thicknesses.

The U-Net++ architecture was utilized for retinal vasculature extraction in a study by the authors in [17]. Extracted features were then used to predict diabetic retinopathy in the subsequent stage. The same task was achieved using an encoder enhanced atrous U-Net by the researchers in [18]. An enhanced U-Net was employed for retinal vasculature extraction by the researchers in [19]. For the same task, three deep learning models, including SegNet, U-Net, and Convolutional Neural Network, were utilized by the researchers in [20]. Among these, SegNet was found to be the most effective. A deformable convolutional network was joined with U-Net architecture to perform retinal vasculature extraction by the researchers in [21]. Another context-involved U-Net approach was employed for retinal vasculature extraction by the researchers in [22]. The extraction of thinner vessels was improved by using patch-based loss weight mapping.

Aujih et al. [23] conducted a study using the U-Net model for retinal vasculature extraction. They used dropout and batch normalization with different settings, finding that batch normalization accelerated learning up to the thirtieth epoch. Additionally, the same study used Inception-V1 to understand the impact of retinal vasculature extraction on diabetic retinopathy classification. The U-Net architecture with region merging was used by the researchers in [24] for retinal vasculature extraction.

In another study by the authors in [25], a backpropagation neural network was employed to achieve retinal blood vessel segmentation, resulting in reduced operation time and improved accuracy. Deng and Ye [26] used a new model called D-MNet, having multi-scale attention and a residual mechanism along with a pulse-coupled neural network for achieving the same task.

Retinal blood vessel segmentation using a multi-encoder decoder architecture having two encoders was performed by the researchers in [27]. Yadav [28] used a dual-tree discrete Ridgelet transform (DT-DRT) to extract features within the Region of Interest in fundus images. Subsequently, a U-Net was utilized to achieve retinal vasculature extraction. Samuel and Veeramulai [29] achieved the same task using a multilevel deep neural network. Feature extraction was performed with VGG-16.

Wu et al. [30], used a new network called NFN+ for retinal vasculature extraction. This *NFN*+ model was characterized by a special cascaded architecture that included connections between networks. These connections facilitated the accurate segmentation of thick and thin retinal blood vessels. Yan et al. [31] used a three-phase deep learning model. This model sequentially extracted thick vessels, followed by thin vessels, and ultimately combined them. This approach yielded the successful extraction of vessels with varying thicknesses. In the work by the authors of [32], a multi-scale Convolutional Neural Network featuring attention mechanisms (MSCNN-AM) was utilized for retinal vasculature extraction. This technique involved utilizing various dimensions for segmentation. To enhance the effectiveness of capturing global and multi-scale vessel data, atrous separable convolutions with different dilation rates were employed. In the same context, some authors used a Generative Adversarial Network (GAN) to segment retinal vasculature. For example, the authors in [33] proposed a conditional pix2pix GAN for segmenting retinal vessels, while in [34] the authors proposed a GAN-based model with an adapted UNet to segment retinal data. In [35], the authors proposed a GAN-based model named M-GAN with an M-generator while two encoder-decoder networks were exploited.

Table 1 summarizes some of the methods that were reviewed in this section.

## 3. Proposed Method

### 3.1. Data Augmentation

All three datasets were small and hence were subjected to various data augmentation techniques. These techniques included Horizontal Flipping, Vertical Flipping, Elastic Transform, Grid Distortion, and Optical Distortion. Horizontal flipping involved flipping the image along the Y-axis, while vertical flipping flipped along the X-axis. Elastic Transformations, Grid Distortion, and Optical Distortion were each applied with two different sets of parameters. Albumentations, an open-source library, was utilized for performing the data augmentation. Among the augmentation methods used, Elastic Transformation and Grid Distortion are particularly renowned for medical images.

### 3.2. GAN-Based Retinal Vasculature Segmentation

**Figure 2:**
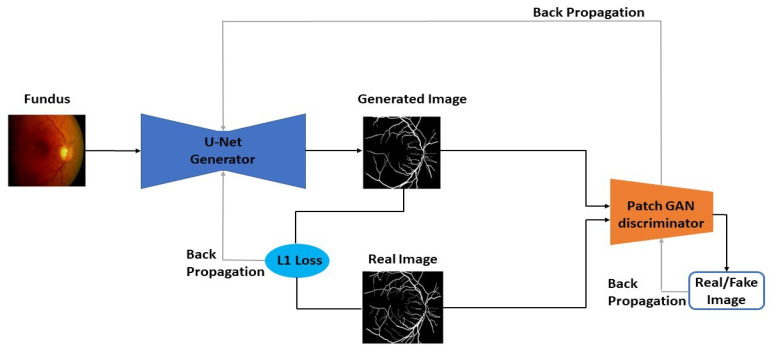
Flowchart of the GAN model.

In this study, a new type of Pix2Pix Generative Adversarial Network (GAN) was employed. Initially introduced by Ian J Goodfellow in 2014, this architecture comprises two sub-models, the Generator as well as the Discriminator. These models compete against each other, with the Generator generating data samples and the Discriminator attempting to differentiate between real and generated data. Training continues until the Discriminator is unable to differentiate between the two. Figure 2 shows the Pix2Pix GAN architecture used in this study.

The Generator network receives a fixed-length random seed noise or latent vector, which it uses to produce an image. This latent vector serves as the foundation of the generative process. The resulting image and real images are fed into the Discriminator for discrimination. After training, a multi-dimensional vector space called latent space is created, representing latent variables that cannot be directly observed but are crucial for the problem domain, resembling points in it. The latent space captures high-level concepts of the unprocessed data, and the Generator interprets points in this space to produce new outputs.

The Discriminator functions as a classification model, distinguishing between real (from the training data) and generated samples. The Generator and Discriminator losses are monitored during training, with the goal of minimizing the Discriminator loss. As training progresses, the Discriminator becomes better at distinguishing real from fake, and the Generator becomes more proficient at generating realistic data. When convergence is reached, the Generator can produce nearly realistic data, and the Discriminator outputs ½ for all inputs, rendering it dispensable after training.

GANs find applications in various domains, such as generating 3D objects, image processing, traffic monitoring, texture transfer, and more [36]. One crucial application is Image Translation, which involves transforming an input image into an output image.

Different types of GANs exist, including DCGAN, cGAN, Cycle GAN, and Info GAN. DCGAN uses deep convolutional nets and transposed convolutional networks for upsampling images. cGANs allow the use of class labels to condition the GAN, which makes it suitable for image-to-image translation. Cycle GAN can perform similar tasks but with the ability to learn mappings between images using unpaired datasets. Info GANs can learn interpretable and meaningful representations. In this study, a Pix2Pix GAN was used, which is a special case of cGAN, widely used for image-to-image translation experiments.

Out of a perceived image *x* as well as a random noise vector *z*, a cGAN can understand a mapping to an output image *y* represented as G:x,z→y [37].

The following denotes the loss function of a cGAN [37]:

(1) $$\begin{array}{c} L_{cGAN}\left(G,D\right)=E_{\left(x,y\right)}\left[\log D\left(x,y\right)\right]+\\ E_{\left(x,z\right)}[\log\left(1-D\left(x,G\left(x,z\right)\right)\right] \end{array}$$

In this, the generator *G* tends to reduce the aforementioned function in contradiction of the discriminator *D*, which tends to increase it. To calculate the implication of conditioning *D*, an unconditional variant is used in the loss for GAN as seen below [37]:

(2) $$\begin{array}{c} L_{GAN}\left(G,D\right)=E_{\left(y\right)}\left[\log D\left(y\right)\right]+\\ E_{\left(x,z\right)}[\log\left(1-D\left(G\left(x,z\right)\right)\right] \end{array}$$

The Generator in the Pix2Pix GAN uses a Resnet between upsampling and downsampling operations, forming a UNet architecture. Additionally, an L1 loss function is introduced in *G* to minimize blurring as follows [37]:

(3) $$\begin{array}{c} L_{L1}\left(G\right)=E_{\left((x,y,z)\right)}\left[{\left||y-G(x,z))|\right|}_{1}\right] \end{array}$$

The Discriminator is a patchGAN with a 70 × 70 patch size. The final loss function of the Pix2Pix GAN is denoted by a formula involving the cGAN loss and the L1 loss, with a hyperparameter λ determining the weight of the L1 loss function [37] as below:

(4) $$\begin{array}{c} \;\;G^{*}=arg\;{min}_{G}\;{max}_{D}L_{cGAN}\left(G,D\right)+\lambda L_{L1}\left(G\right) \end{array}$$

## 4. Experimental Results

In this section, we present the experimental results using the proposed method on three datasets. In the experiment using the ARIA dataset, a total of 1287 images were used which is quite large, leading to a high number of trainable parameters. To handle this, a laptop with GPU capabilities was used, featuring an Intel Core i7 processor as well as an NVIDIA GEFORCE graphics card.

We trained our Pix2Pix GAN using the PyTorch framework in a Python 3.9 environment. The Adam optimizer, having an initial learning rate set at 0.0002 along with *L*1 loss (λ) set to ten, was used. We trained the model for 100 epochs.

### 4.1. Datasets

The segmentation evaluation was performed on the following three datasets including ARIA, DRIVE, and HRF.

The ARIA (Automated Retinal Image Analysis) dataset comprises 143 fundus images annotated for blood vessel segmentation [38]. Each image has dimensions of 768 × 576 pixels and includes images of both left and right eyes. The dataset was gathered during the period 2004 to 2006 by St. Paul’s Eye Unit, Liverpool, UK, from male as well as female adults. It consists of three groups: the control group with 61 images, the diabetic retinopathy group with 59 images, and the age-related macular degeneration (AMD) group with 23 images. Two different graders, denoted as “SS” and “BD”, annotated the images, and the labels from grader “BD” were used in this experiment. Eighty percent of the data was used to train and the rest to test the model.

The DRIVE dataset (Digital Retinal Images for Vessel Extraction) contains fundus images acquired through a DR diagnosis initiative in the Netherlands [39]. It comprises forty images, separated equally into train and test sets. A Canon camera at forty-five degrees field of view was utilized to capture these images, having a resolution of 584 × 565 pixels. In the training set, each image is annotated by a single expert. In contrast, the testing set contains two annotations for each image, performed by two different graders. To assess the proposed method on this dataset, we use the annotations provided by the first grader. The same training and testing sets provided in the dataset were used in this experiment.

The High-Resolution Fundus or HRF dataset consists of forty-five high-resolution color fundus images having a size of 3504 × 2366 pixels [40]. The images present in it are separated into three categories consisting of healthy, DR, and glaucomatous with 15 images each. All images are provided with binary gold standard vessel segmentation. Eighty percent of the data was used to train and the rest to test the model.

### 4.2. Evaluation Metrics

To evaluate the model’s performance, seven metrics were employed, which include Accuracy, Sensitivity, Specificity, Dice Coefficient, Jaccard’s Coefficient, Precision, as well as Matthews Correlation Coefficient (MCC). All of the metrics chosen in this study are commonly used in segmentation tasks. To understand the formulas used for calculating these metrics, some terms need to be defined including TP or “True Positives” which denotes the correctly generated retinal blood vessel pixels. TN or “True Negatives” denotes the rightly generated background pixels. FP or “False Positives” denotes the background pixels falsely acknowledged as retinal blood vessel pixels. FN or “False Negatives” denotes the retinal blood vessel pixels falsely identified as background pixels.

The following formulas were used to calculate the mentioned metrics in this study:

**Accuracy**: Pixel-wise accuracy measures how many pixels the model classifies correctly.

(5) Accuracy=(TP+TN)/(TP+FP+FN+TN)

**Sensitivity**: This metric measures the rate of actual pixels generated as retinal blood vessels among all generated pixels that are actually retinal blood vessel pixels.

(6) Sensitivity=TP/(TP+FN)

**Dice** Coefficient(Sorenson Index/FMeasure): An important metric used in image segmentation, representing a special overlap index.

(7) Dice=2∗TP/(2∗TP+FP+FN)

**Specificity**: This metric measures the rate of actual pixels generated as background pixels among all generated pixels that are actually background pixels.

(8) Specificity=TN/(TN+FP)

**Jaccard**’s Coefficient: This metric indicates the similarity between two images.

(9) Jaccard=Dice/(2−Dice)

**Precision**: This metric denotes the rate of actual pixels generated as retinal blood vessels to the entire count of pixels projected as retinal blood vessels.

(10) Precision=TP/(TP+FP)

**MCC**: MCC estimates the distance between the actual values and projected values.

(11) $$\begin{array}{c} \mathrm{MCC}=(\mathrm{TP}*\mathrm{TN}-\mathrm{FP}*\mathrm{FN})/\mathrm{sqrt}((\mathrm{TP}+\mathrm{FP})*\\ \left(\mathrm{TP}+\mathrm{FN})*(\mathrm{TN}+\mathrm{FP})*(\mathrm{TN}+\mathrm{FN})\right) \end{array}$$

### 4.3. Evaluation and Discussion

**Figure 3:**
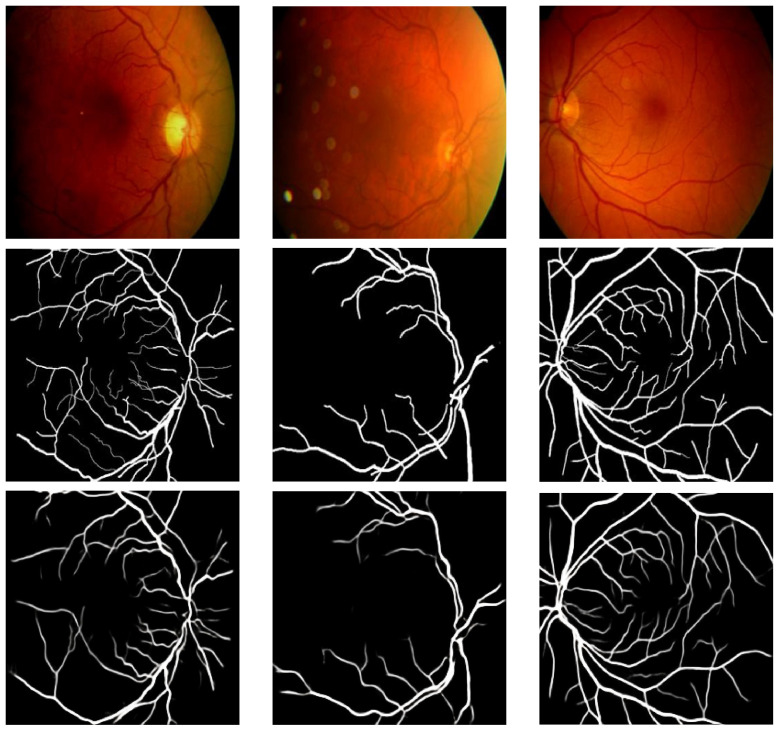
Results of Pix2Pix GAN on the ARIA dataset (first row: fundus images; second row: groundtruth; and third row: results).

**Figure 4:**
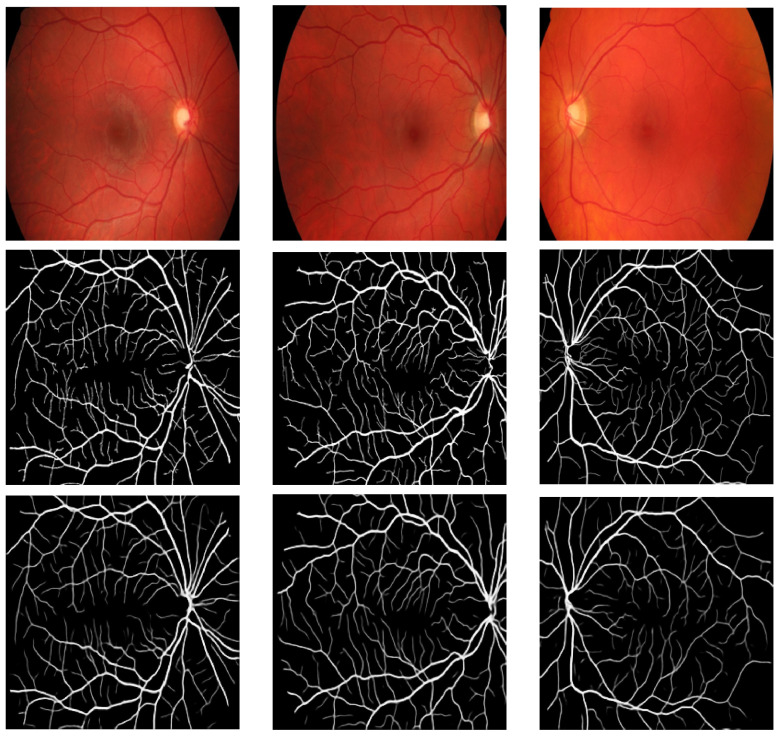
Results of Pix2Pix GAN on the HRF dataset (first row: fundus images; second row: groundtruth; and third row: results).

**Figure 5:**
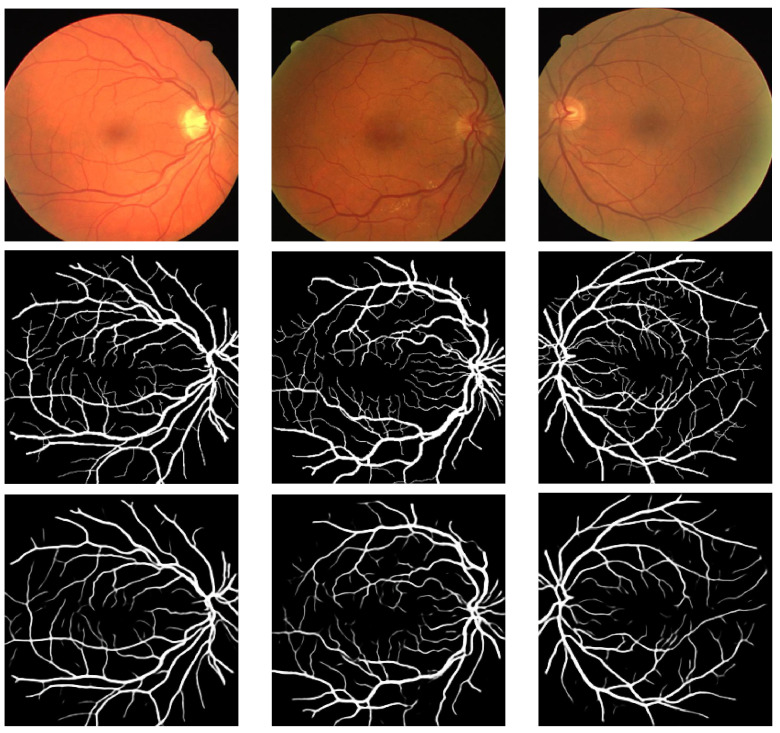
Results of Pix2Pix GAN on the DRIVE dataset (first row: fundus images; second row: groundtruth; and third row: results).

The values attained for the different metrics presented in the preceding sub-section are shown in Table 2. We also present some visual results in Figure 3, Figure 4 and Figure 5. Table 2 presents a contrast of the results attained by our model with previous techniques that utilized the ARIA, DRIVE, and HRF datasets for retinal vasculature segmentation.

Table 2 reveals that the GAN applied to all three datasets achieved values above 0.942 for all seven metrics calculated in this study. Higher values closer to one indicate better results. Thus, these metrics demonstrate the GAN model’s strong performance on all three datasets, particularly for retinal vasculature extraction. Furthermore, visually comparing the results with the ground truth presented compelling and appealing outcomes. The highest values for all metrics were obtained on the HRF dataset except the one obtained for Sensitivity. The highest value for Sensitivity was obtained on the DRIVE dataset.

Table 2 shows that our model performed better than other methods used in the comparison. Specifically, we compared our results on the ARIA dataset with three methods that utilized the same dataset: Azzopardi’s method [44], Kar’s method [41], and Prajna’s method [43]. Vostatek et al. [42] evaluated Azzopardi’s traditional method [44] using the ARIA dataset. Kar et al. [41] employed a Deep Neural Network (DNN) for retinal vasculature segmentation. Similarly, Prajna and Nath [43] tackled the same task by combining a Multi-Scale Residual CNN with GAN. Compared with three other studies chosen for comparison, the GAN model yielded superior results regarding Accuracy, Sensitivity, Dice, Jaccard, and Precision metrics on this dataset. Additionally, the model obtained the second-highest value for Specificity.

The results obtained on the DRIVE and HRF datasets were compared alongside three studies by the researchers in [20,22,41]. As mentioned earlier, the researchers in [41] employed a DNN for retinal vasculature segmentation. Elaouaber [20] used three deep learning models, which included SegNet, U-Net, and CNN, to achieve the same. They obtained the best results using SegNet, whereas the researchers in [22] used a context-involved U-Net approach for retinal vasculature extraction. Table 2 shows that on the DRIVE dataset, we achieved the highest values for Accuracy, Sensitivity, Dice, MCC, and Precision when compared with the other three studies. Regarding the HRF dataset, we could obtain the highest values for Accuracy, Dice, and Precision in the comparison. Moreover, we could attain the second-best values for Sensitivity and Specificity.

## 5. Conclusions and Future Work

We could successfully use the GAN model on the HRF dataset with an accuracy of 0.983, a sensitivity of 0.973, as well as a specificity of 0.992 for retinal vasculature extraction. The results achieved on the DRIVE and ARIA datasets were also appealing. Notably, these favorable outcomes were attained despite using smaller datasets. In future work, we will perform diabetic retinopathy lesion segmentation using similar deep-learning methods.

## Figures and Tables

**Table 1:** Previous methods for diabetic retinopathy retinal vasculature segmentation.

Study	Method	Dataset(s)	Year
Gargari et al. [17]	U-Net++	DRIVE, MESSIDOR	2022
Roychowdhury et al. [11]	Gaussian mixture model classifier	DRIVE, CHASEDB1, STARE	2014
Fan et al. [10]	Image matting	DRIVE, CHASEDB1, STARE	2018
Memari et al. [9]	Fuzzy c means clustering	DRIVE, CHASEDB1, STARE	2019
Zhang et al. [22]	U-Net	DRIVE, CHASE-DB1, STARE, HRF	2022
Atli and Gedik [16]	Sine-Net	DRIVE, CHASEDB1, STARE	2021
Sathananthavathi et al. [18]	U-Net	CHASE DB1, DRIVE, STARE, HRF	2021
Deng and Ye [26]	D-MNet	CHASE DB1, DRIVE, STARE, HRF	2022
Elaouaber et al. [20]	Multiple DL models	DRIVE, CHASE-DB1, HRF	2022

**Table 2:** Performance comparison of results with previous methods on the ARIA, DRIVE, and HRF datasets. The **bold** font represents **first** place.

Dataset	Method	Accuracy	Sensitivity	Specificity	Dice	Jaccard	MCC	Precision
DRIVE	Kar et al. [41]	0.974	0.894	0.988	-	-	-	0.875
	Elaouaber et al. [20]	0.977	0.967	**0.996**	0.957	-	-	-
	Zhang et al. [22]	0.957	0.785	0.982	0.82	-	0.798	0.864
	Popescu et al. [33]	0.921	0.834	0.960	-	-	-	0.948
	Yue et al. [34]	0.970	0.833	0.985	-	-	-	-
	Park et al. [35]	0.970	0.834	0.983	-	-	0.826	0.834
	**Proposed**	**0.978**	**0.975**	0.981	**0.978**	0.956	**0.956**	**0.98**
HRF	Kar et al. [41]	0.977	0.889	0.985	-	-	-	0.8
	Elaouaber et al. [20]	0.98	**0.98**	**0.995**	0.969	-	-	-
	Zhang et al. [22]	0.96	0.85	0.971	0.82	-	-	-
	Park et al. [35]	0.967	-	-	-	-	0.784	-
	**Proposed**	**0.983**	0.973	0.992	**0.982**	0.965	0.966	**0.992**
ARIA	Kar et al. [41]	0.963	0.718	**0.984**	-	-	-	0.795
	Vostatek et al. [42]	0.94	-	-	-	-	-	-
	Prajna and Nath [43]	0.925	0.566	0.961	0.649	0.48	-	-
	**Proposed**	**0.971**	**0.974**	0.969	**0.97**	**0.942**	0.943	**0.966**

## Data Availability

The datasets used in this paper are available online including DRIVE dataset https://drive.grand-challenge.org/ (accessed on 30 March 2023), HRF https://figshare.com/articles/dataset/A_robust_technique_based_on_VLM_and_Frangi_filter_for_retinal_vessel_extraction_and_denoising/5879803 (accessed on 30 March 2023), and ARIA [38].
